# Publication analysis in Bay for Bengal Initiative for Multi-Sectoral Technical and Economic Cooperation Nations

**DOI:** 10.12688/f1000research.52286.1

**Published:** 2021-06-28

**Authors:** Manthan Janodia, Aparna I. Narayan, Santhosh Krishnan Venkata, Bharti Chogtu

**Affiliations:** 1 Manipal College of Pharmaceutical Sciences, Manipal Academy of Higher Education, Manipal, India; 2 UniARC services LLC, Manipal, India; 3 Manipal College of Dental Sciences, Manipal Academy of Higher Education, Manipal, India; 4 Manipal Institute of Technology, Manipal Academy of Higher Education, Manipal, India; 5 Kasturba Medical College, Manipal Academy of Higher Education, Manipal, India

**Keywords:** BIMSTEC, Collaboration, Scopus, Field Weighted Citation Impact

## Abstract

**Background:** Research output provides an insight into the development of the scientific capability of a country. Budget allocation for research and development (R&D) is directly proportional to the research output of a country. While developed countries spend a significant percentage of their GDP on R&D, developing countries do not have enough resources to invest in R&D. Countries in the Bay of Bengal Initiative for Multi-Sectoral Technical and Economic Cooperation (BIMSTEC) Nations has received significantly less attention from outside the region in studying R&D and research publication scenario of the region. The research output of BIMSTEC countries was analyzed using various metrics in this paper.

**Methods:** Data on citation per paper, Field Weight Citation Impact (FWCI), paper per researcher, collaborative publications, and output in top 10 percent journals was extracted from one of the largest abstract and citation database of peer-reviewed literature, Scopus and its affiliate SciVal, for a period of 6 years between 2012-2017. Percentage of R&D spend, researchers per million population, and total scientific output were extracted from World Bank data.

**Results:** India and Thailand have a higher quantum of publications compared to other countries. Subjects like clinical, technology, Computer Science have a larger publication number as compared to other subject areas like Social Science, Arts, Education, Law, and Physiology. The researcher population and research spend of a nation have an evident implication on the publication though no direct relation can be derived.

**Conclusion:** Huge disparities in terms of percentage of research spent, research output, papers per researcher, and output with national and international authorship differ for countries. Higher research spent and publication count are not positively correlated with better FWCI.

## Introduction

The formation of the South Asian Association of Regional Conference (SAARC) was a landmark step in developing economic and regional integration in this area but the outcomes in terms of strengthening regional cooperation even after three decades were not satisfactory. ^1^ This forced the regional members to look for an alternative option and the search led to the commencement of the Bay of Bengal Initiative for Multi-Sectoral Technical and Economic Cooperation (BIMSTEC). Initiated as BIST-EC in June 1997 with the inclusion of Bangladesh, India, Sri Lanka, and the Thailand Economic Corporation, saw Myanmar being included late in the same year. In 2004, Nepal and Bhutan also became members of this group and the group was renamed to its present name BIMSTEC. ^2^ It fosters socio-economic linkages between South Asia and South-East Asia and includes 1.5 billion people constituting around 22% of the global population with a combined Gross Domestic Product (GDP) of 2.7 trillion economy. Elementally, it started with cooperation in six sectors: trade, technology, energy, transport, tourism, and fisheries. Subsequently, other sectors were incorporated and there was a commitment for active collaboration in training and research facilities in fields of common interest. The member states felt that knowledge sharing, research and development, and capacity building in various fields like climate change and public health among the BIMSTEC nations can provide solutions for problems of the future. ^3^

The bilateral and sub-regional projects within BIMSTEC nations were a uniting force to bring this region under one economy. The 15 ^th^ BIMSTEC ministerial meeting held in Nepal in the year 2017 agreed upon enhancing cooperation in different fields like economic integration connectivity and mutual support in disaster management. This meeting also decided upon implementing policies aligning with the United Nation’s Sustainable Development Goals (SDGs), which addresses global concerns like climate change, environment, and reducing poverty. Also, India-Bangladesh Joint research in oceanography was discussed as a bilateral agreement. ^4^

BIMSTEC nations face diverse challenges to excel in the field of research and development. One of the many hurdles faced by developing countries like Sri Lanka is inaccessibility to research papers, which in turn decreases the scientific output. ^5^ The Sri Lankan Government has launched initiatives to promote Research and Development, which include a presidential award at the national level for outstanding researchers based on data from the Science Citation Index (SCI), research allowance, and promotion benefits. ^6^ On comparing the research output amongst different streams in Sri Lanka, Science and Medicine tops the chart followed by Engineering, Agriculture, and Management ^7^ as mentioned in the National Research Council of Sri Lanka. In ten years (2000-2010) certain bodies like the National Research Council focused on the funding of researchers. ^8^

In 2008, the National Science, Technology and Innovation policy (STI) was established in Thailand. National STI Master Plan 2012-2021 aims at collaboration between private sectors, research institutes, and academics. Thailand research fund is a leading funding agency and does not come under the umbrella of government bureaucracy and thus supports research efficiently. ^9^ Secretary General of STI has put forth that the investment in research and development has increased by 36% in 2018 as compared to the previous year. The proportion of researchers is increasing over the years at an annual rate of 4/10,000 population. The government is encouraging private investors to invest in research and development by providing funding for startups, expanding economic innovation zones, and favorable funding conditions. In Thailand, artificial intelligence related research is carried out after 2011 and two exemplary projects were on Speech Technologies and Health Car Knowledge Engineering, and Agricultural Cyber Brain. With Smart Thailand 2020, the country is trying to move ahead in the field of research. ^10^

Low-income countries like Nepal lack national commitment for research and a dearth of resources to conduct research. The poor state of higher education has an adverse effect on the volume and growth of research and the prevailing political and economic situations contribute to the same. ^11^ Earlier, scientometric analysis suggested areas like medicine, agriculture, and biological sciences were highly productive. ^12^ In the years 2015 and 2016 there was a sudden upsurge of publications after the devastating Gorkha Earthquake that set off the collaborative research in seismology, engineering, geology, civil engineering, and other fields. ^11^ Since 2017, smaller countries like Nepal and Sri Lanka have increased their research output by engaging in international collaborations and by this they are gaining expertise in addition. ^13^

After 2011, the research environment in Myanmar has changed with facilities for international researchers to carry out research in Myanmar by relaxing travel restrictions, increased research funding from the Myanmar government and other international organizations like World Bank, ^14^ bibliometric analysis of social sciences and humanities from 2005 to 2014 in India show that research articles increased from 62.1% to 82.9%, collaborative research was done routinely and it received more citations. ^15^ From 2000 onwards there has been an exponential growth in publications in India. But this increase in volume does not commensurate with the citations of Indian research. ^16^ A large part of research in India is published in regional or local languages which are not included by the indexing agencies. ^15^

As per Web of Science (WoS) data, India published 4780 highly cited papers from 1989-2015. Papers from international collaborators received more citations as compared to others. In this period, the United States of America was the major collaborator accounting for 22.29% of highly cited papers and the average citation was 238.92 per paper. Collaborative papers with the US were followed by Germany and China. ^17^ In papers published in the research field from 2009-14, India stands at fifth position in Chemistry, eighth position in Physics, and ninth position in Material Science. In the subject area of Computer Science, which is considered a thrust area throughout the globe, India has improved its position from twelfth to third. However, India has still a long way to go in Medical and Biosciences research. ^18^

South Asian countries like Bhutan, Nepal, India, and Sri Lanka have common health challenges like chronic conditions, infectious diseases, and injuries. Health systems also encounter a lack of social accountability making these countries benefit from collaborative health research. ^19^ Myanmar, the land bridge between ASEAN and India has a vital role in shaping the economic, political, and security environment in the region. ^20^ With the rising interest on the BIMSTEC nations, this paper aims to discuss the outcomes of the BIMSTEC nations as a group in terms of research output such as the number of publications, citations per paper, spend on research and development (R&D), Field Weighted Citation Impact (FWCI) across different subject areas and international collaborations. Analyzing the aforementioned outcomes of the BIMSTEC countries for the period 2012-17.

### Problem statement

**Figure 1.**
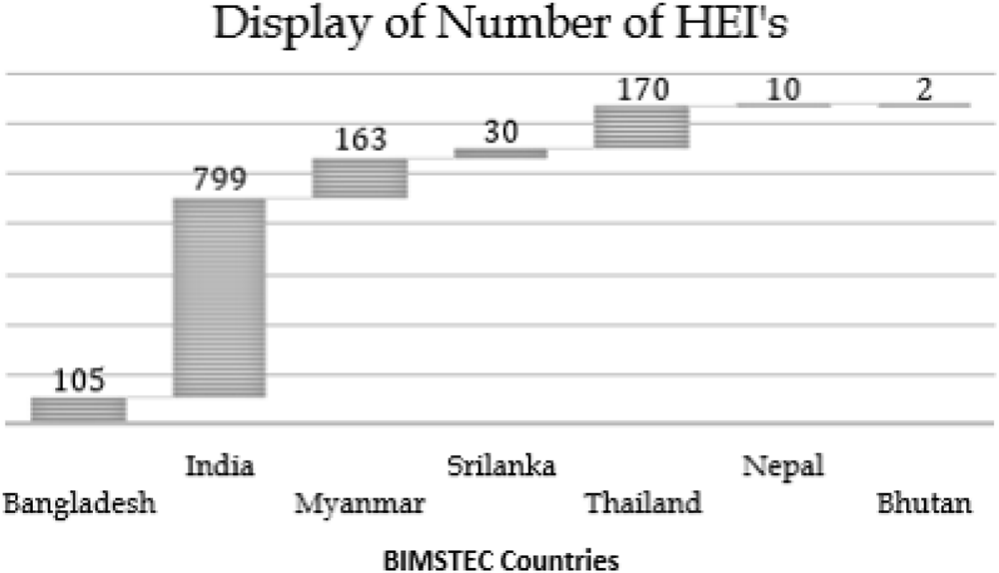
Number of higher education institutions in BIMSTEC countries as of 2020.

**Figure 2.**
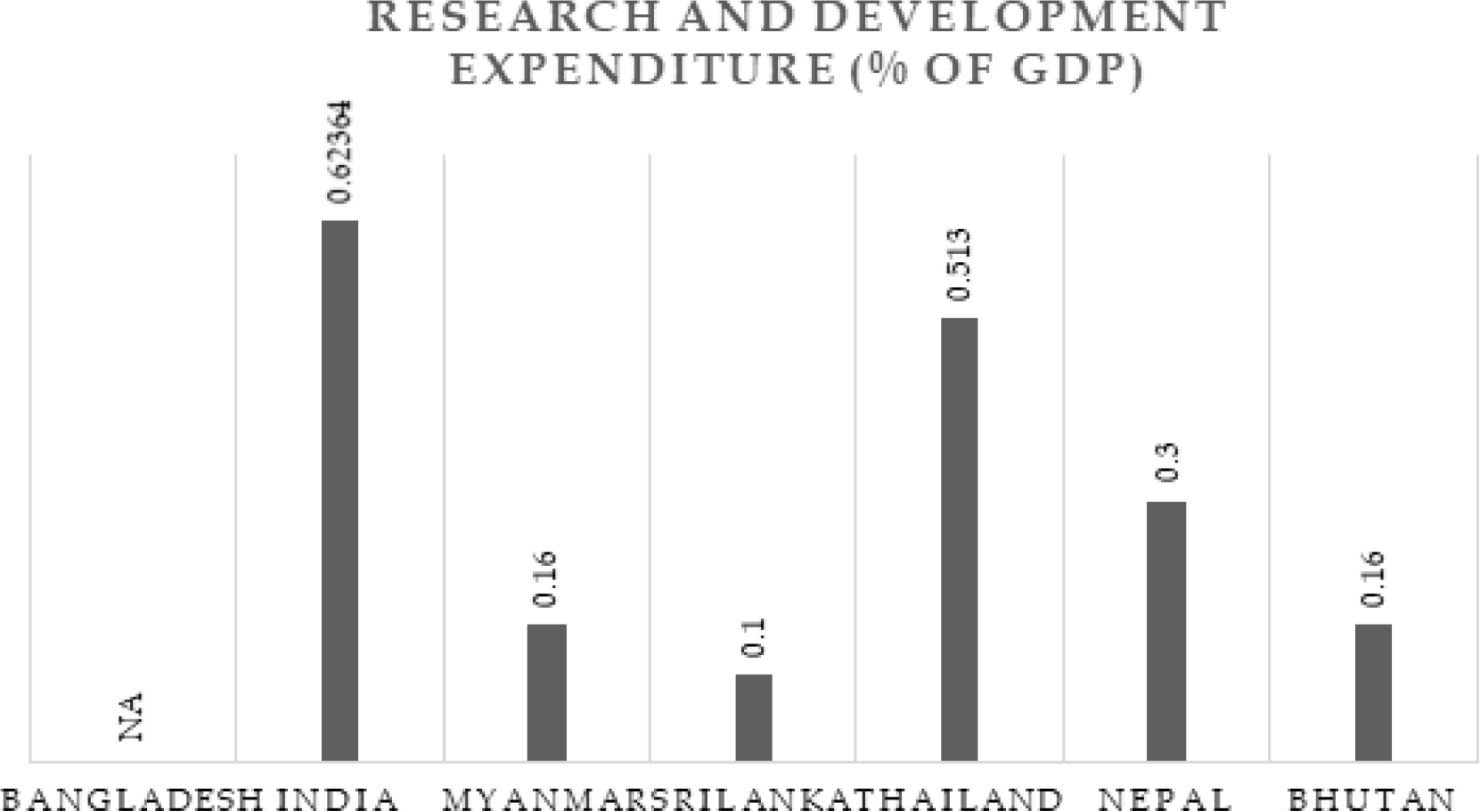
Research and development expenditure BIMSTEC countries (% of GDP) (2012-17).

**Figure 3.**
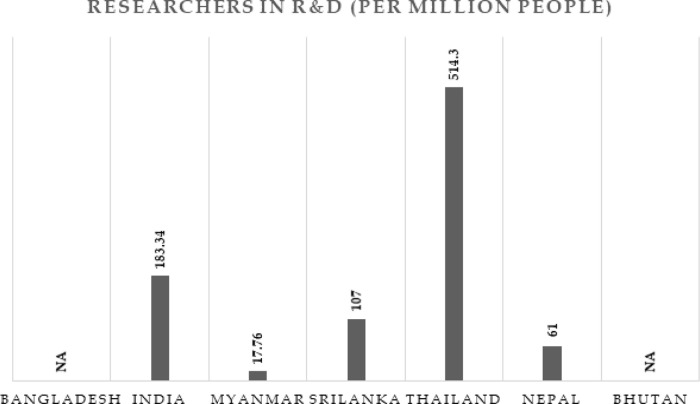
Researchers in Research and Development (per million people) in BIMSTEC countries (2012-17).

Research outcomes of a country are often associated with the quantity and quality of higher education institutions (HEIs). This paper attempts to analyze the research outcomes of the BIMSTEC countries for the period 2012-17. A comparative representation of the number of HEIs in each of the BIMSTEC countries is shown in Figure 1. Based on the data it can be seen that India has the highest number of HEIs with 799, followed by Thailand with 170, then Myanmar with 163, Bangladesh with 105, Sri Lanka with 30, Nepal with 10 and Bhutan with 2. These numbers would also have a direct relation to the population of each of the countries. To further understand the research parameters of BIMSTEC countries data with regard to percentage GDP spent on research and researcher population per million is plot in Figures 2 and 3, respectively. The data is extracted from the World Bank report on 4 ^th^ April 2020.

It is to be noted that the average percentage of GDP spent on research is highest in India, which is around 0.62%, followed by Thailand which is 0.51%, then Nepal which is 0.3%, Bhutan and Myanmar spend around 0.16%, and the least spent is from Sri Lanka with 0.1%. Bangladesh has not reported any data with regard to research spent in the World Bank repository. Data corresponding to each research population is derived from World Bank repository as on 4 ^th^ April 2020 ( Figure 3). It can be seen that Thailand has the greatest number of research population per million with is around 514, followed by India with 183, Sri Lanka with 107, Nepal with 61, and Myanmar with 17. Data related to Bangladesh and Bhutan were not available in the World Bank data.

From the data, it can be seen that the source for research in terms of researcher population and GDP spending is higher in countries like India and Thailand. Bhutan, Myanmar, Sri Lanka, Nepal, and Bangladesh have a lesser researcher population and expenditure in terms of GDP percent. In this paper, a detailed analysis of the research outcomes is presented which would give a fair idea of the correlation of the research sources of those countries. The paper focuses on the research outcomes in terms of research publications, which includes article, review papers, conference proceedings, book chapters, books, case reports, etc. published in Scopus indexed publications. Does publication performance/quantum of publication have any relation with research spend or researcher population of the country? An attempt is therefore made to understand the publication outcome of BIMSTEC countries during the period 2012-17.

## Methods

For the analysis of publication outcome of BIMSTEC countries, data was obtained from one of the largest abstract and citation database of peer-reviewed literature, Scopus on 3 ^rd^ of March 2020 and its affiliate SciVal on 3 ^rd^ March 2020. These databases provide options to extract various scientific parameters through different permutations and combinations for each country as well as for the region, in the present work the data corresponding to seven BIMSTEC countries namely Bangladesh, India, Myanmar, Sri Lanka, Nepal, Thailand and Bhutan is obtained for the period 2012 to 2017. With respect to this reported work publications, research articles, reviews, conference proceedings and book chapters were only considered. Case reports, letter to editors, technical reports and brief/short communications were not considered. The publication types for inclusion and exclusion were considered based on the uniformity of data across all the countries and relevance to the reported study. Moreover, publication of articles, reviews, conference proceedings and book chapters are produced in higher volumes and have considerable higher citation metrics as compared to the other types.

World Bank Indicators for identifying research expenditure as a percentage of GDP, researchers per million population was extracted on 4 ^th^ April 2020. Country-wide data corresponding to these two parameters are taken for the period 2013-2017 and the average value of these data for the period is chosen as the figure for all computations.

BIMSTEC countries were selected for analysis as it is one of the leading associations for regional cooperation in South East Asia. The analysis of scientific disciplines was based on subject categories as Scopus distributes publications into various subject categories. Around 300 subject categories are divided among the five major subject areas namely Art & Humanities, Engineering & Technology, Life Science & Medicine, Physical Science and Social Science & Management. This paper presents eleven subject groupings that were common for ease of analysis based on subject categories provided by Times Higher Education (THE). These subjects are Arts & Humanities, Business & economics, Clinical, Computer Science, Education, Engineering & Technology, Law, Life science, Physical sciences, Psychology and Social sciences.

### Data analysis

Publication related data for the period 2021-2017 was exported from Scopus to SciVal analytics tool by Elsevier. SciVal is an online tool used to analyze research performance. Data is analyzed on 4 ^th^ of April 2020. Publication data is analyzed for the period 2012-17 across the BIMSTEC countries for eleven subjects mentioned; outcome based on collaborative publication is also studied. Once analyzed data is it was exported to Excel, which was used to represent the data in the form of graph and plots. ^21^

## Results

**Figure 4.**
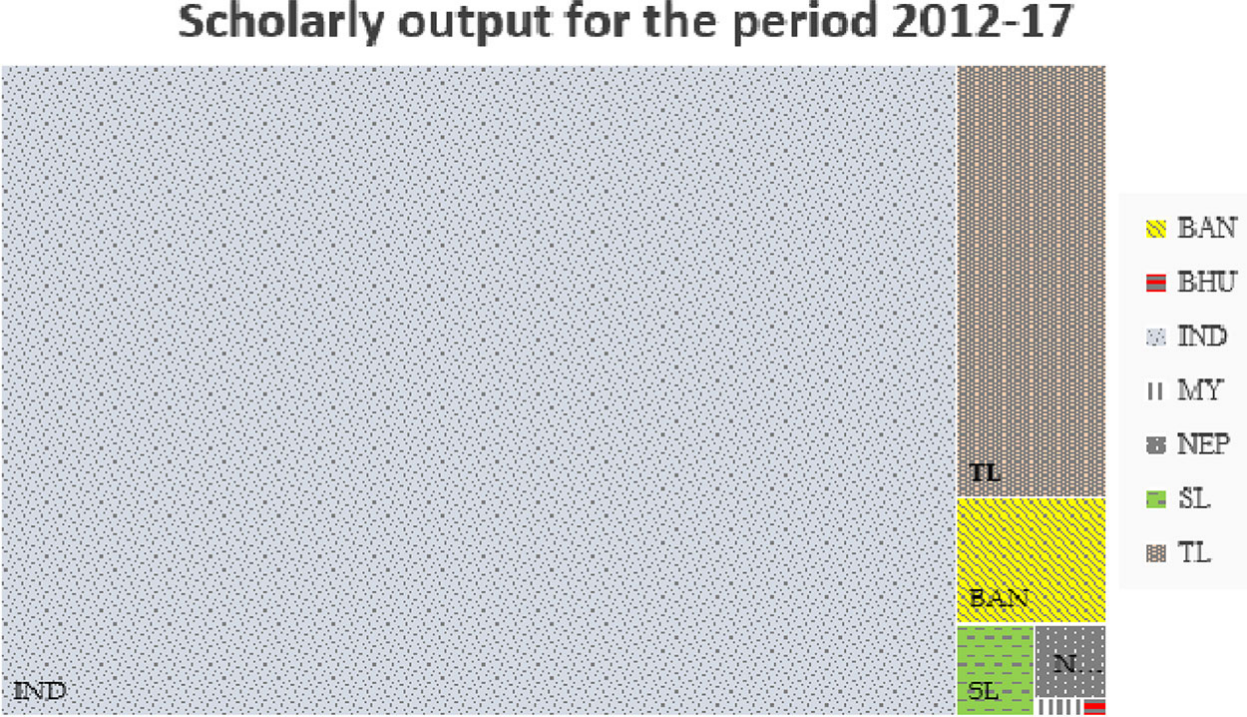
Treemap representation of scholarly output of BIMSTEC countries. India (IND), Thailand (TL), Bangladesh (BAN), Sri Lanka (SL), Nepal (NEP), Myanmar (MY), Bhutan (BHU).

Table 1 shows the scholarly output of each of the BIMSTEC countries in Scopus indexed publication for 2012-17. It can be seen that India has a maximum number of publications with around 0.8 million publication, followed by Thailand, which is around 80 thousand, Bangladesh around 24 thousand, Sri Lanka around 10 thousand, Nepal around 7 thousand, Myanmar with around 1000 publication, and Bhutan has the least number of five hundred. Figure 4 shows the magnitude-wise representation of the scholarly output which can be seen that publications from India constitute almost 80% of BIMSTEC publications, with Thailand being the nearest country with around 10% of its number. Publication outcome and quality of publication shows correlation with researcher population, percentage GDP on research, and the number of HEIs.

**Table 1.** Scholarly outcome in Scopus indexed publication for the years 2012-2017.

Countries	Scholarly output
Bangladesh	24201
Bhutan	518
India	800159
Myanmar	1236
Nepal	6755
Sri Lanka	9067
Thailand	82755

The important measure of research along with the quantity of publication is also quality of the publication. To measure the quality of the publication, the most common parameter considered is the number of citations. But the absolute number of citations is biased across the different subject areas, as the global average of citation per publication is not uniform across the subjects. So, we have considered FWCI as the measure of the quality of research publication. FWCI is a normalized number of citations per paper across the globe where the average citation index for a given subject area across the globe is normalized to one.

**Figure 5.**
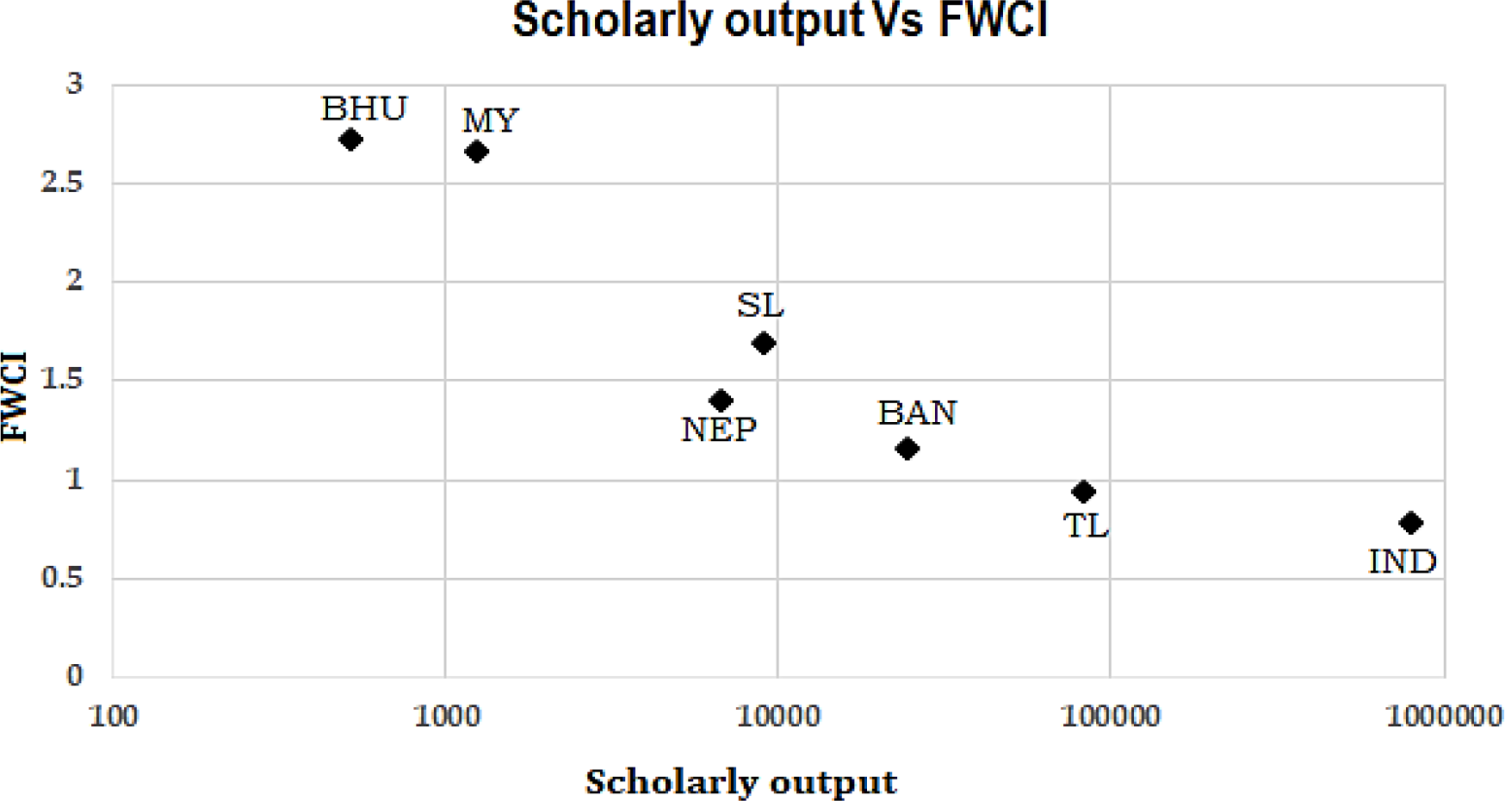
Comparison of FWCI with the scholarly output of BIMSTEC countries. India (IND), Thailand (TL), Bangladesh (BAN), Sri Lanka (SL), Nepal (NEP), Myanmar (MY), Bhutan (BHU).

Figure 5 shows the relation of the average FWCI of the country with respect to its total scholarly output during the period 2012-17. It is seen that publications from Bhutan (2.5) and Myanmar (2.3) have larger FWCI as compared to other BIMSTEC countries, which is surprising looking at the source data of these countries in terms of the number of HEIs or researcher population or research spent. Publications of Thailand and India have the least FWCI of the BIMSTEC countries with around 0.9 and 0.75, respectively. Nepal, Bangladesh, and Sri Lanka have a medium ranged FWCI with around 1.4, 1.2, and 1.7, respectively. From Figure 5 it is evident that the quality vs quantity has an inverse relation in BIMSTEC countries.

### International collaborative publication

To understand the reason for the change in quality index with quantity, we put forth a few other correlated parameters other than the commonly considered parameters like the number of HEIs or research population, and research spent. One such important parameter is the international collaborative publication. The international collaborative publications are those publications which are coauthored by other country researchers.

**Figure 6.**
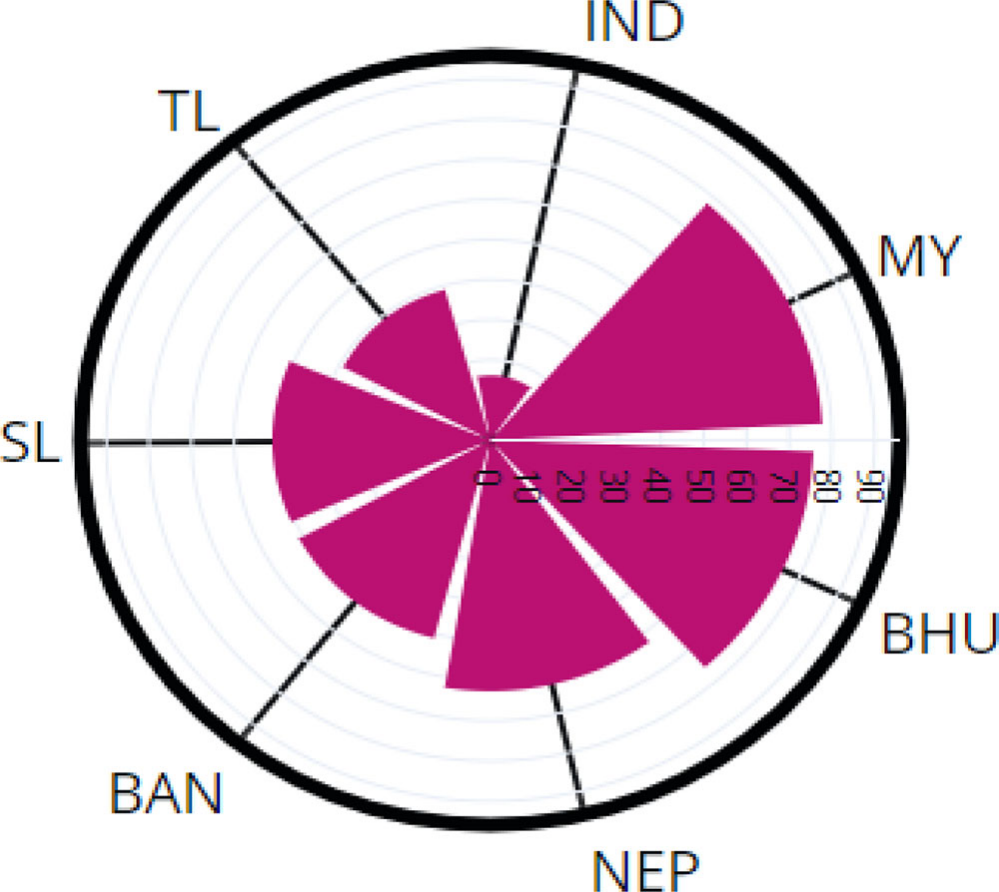
International collaboration publication percentage of BIMSTEC countries. India (IND), Thailand (TL), Bangladesh (BAN), Sri Lanka (SL), Nepal (NEP), Myanmar (MY), Bhutan (BHU).

The percentage of collaborative publication of BIMSTEC countries is shown in Figure 6. A total of 16.1% of publications from India have international coauthors; Thailand, 39%; Sri Lanka, 50.9%; Bangladesh, 51% of publication; Nepal, 62.9%; Bhutan, 75.7%; and Myanmar, 77.8%.

**Figure 7.**
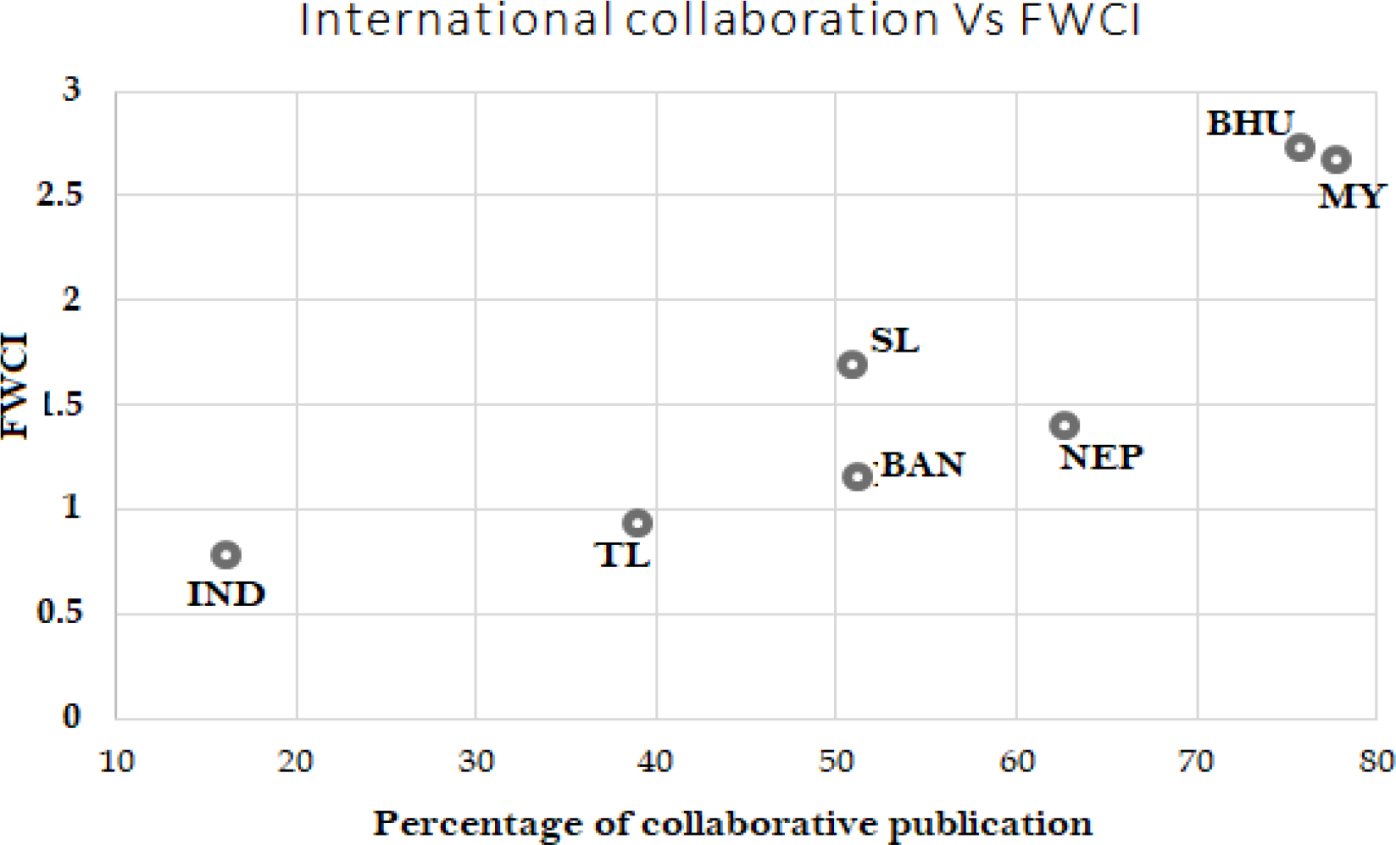
Comparison of FWCI with the percent of international collaborative papers. India (IND), Thailand (TL), Bangladesh (BAN), Sri Lanka (SL), Nepal (NEP), Myanmar (MY), Bhutan (BHU).

To understand the effect of international collaboration on the quality of the publication, a comparison between the percentages of international collaboration to that of FWCI of the nation is indicated in Figure 7. It is seen that countries with a higher number of collaborative publications, like Myanmar (77.8%) and Bhutan (75.7%), have higher FWCI of 2.3 and 2.5, respectively. India with the least collaborative publication of 16.1% has the lowest FWCI of 0.75. The rest of the BIMSTEC countries show similar behavior in the relationship where higher international collaborative publication yield in higher FWCI: Nepal 62.9% international collaborative publications, FWCI of 1.4; Bangladesh 51% international collaborative publications, FWCI of 1.2; Sri Lanka 50.9%, FWCI of 1.7. Therefore, it can be suggested that the quality of publication depends on international collaborations.

**Figure 8.**
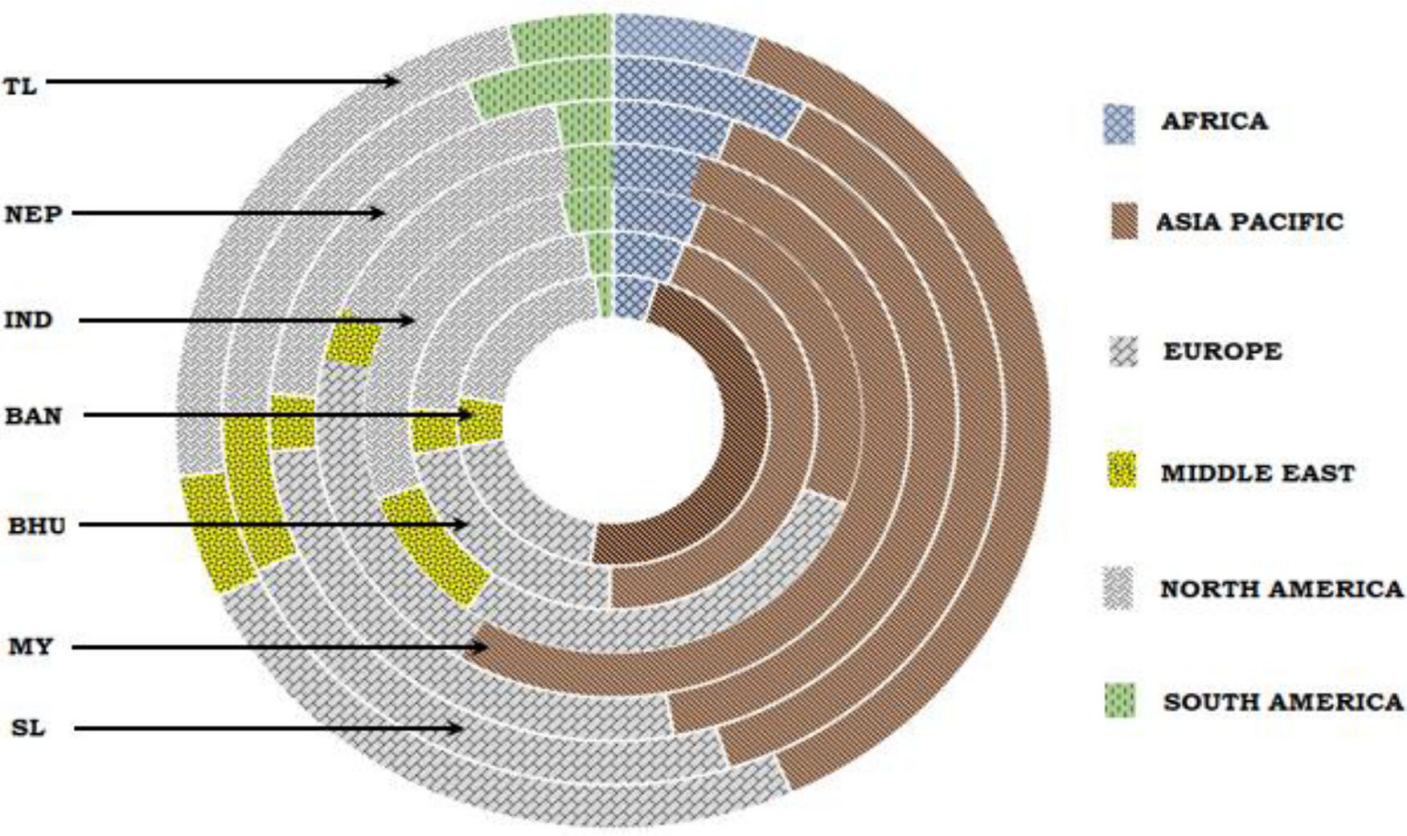
Share of international collaborative publication of BIMSTEC. India (IND), Thailand (TL), Bangladesh (BAN), Sri Lanka (SL), Nepal (NEP), Myanmar (MY), Bhutan (BHU).

The term collaborative publication refers to the coauthored publications from authors affiliated with different countries. An attempt is made in this work to analyze the breakup of coauthored publications in terms of six regions; Africa, Asia Pacific, Europe, Middle East, North America, and South America, to understand the collaborating partners of different BIMSTEC countries. Representation of the distribution of coauthored publications of BIMSTEC countries in the six regions is shown in Figure 8, and the comparison in terms of absolute number is reported in Table 2. It can be seen that all countries, except India, have a large share of collaborative publications within Asia Pacific region whereas India has the highest percent of collaborative publications with Europe, followed by North America. It is also seen that the least percent of collaborative publication by all the BIMSTEC nations is with South America. It is also observed that around 70-80% of the collaborative publication of all the BIMSTEC nations are from the Asia Pacific, Europe, and North American region. The variation in nature of collaboration may be influenced by demographic or political or research interests.

**Table 2.** Comparison of international collaboration publication of BIMSTEC countries.

	Bangladesh	Bhutan	India	Myanmar	Nepal	Sri Lanka	Thailand
Africa	678	32	9114	64	319	588	2220
Asia Pacific	7039	249	37952	711	2324	2636	15680
Europe	2907	122	44296	265	1472	1642	10162
Middle East	818	22	14583	39	162	506	2026
North America	3014	121	41258	209	1173	1322	9633
South America	275	13	5183	35	153	438	1608

### Across subject area

Along with international collaboration, one more parameter that would influence the quality of the publication is the subject area of the research. As per the All Subject Journal Code (ASJC), every journal is mapped to any of the 300+ subject areas, grouped among 11 main subject areas. These subject areas are Arts & Humanities (AH), Business and Economics (BE), Clinical, pre-clinical and health (CH), Computer Science (CS), Education (ED), Engineering and Technology (ET), Law, Life Science (LS), Physical Science (PS), Psychology (PY), and Social Science (SS). A detailed analysis is presented in this section where each of BIMSTEC country’s outcome in terms of the number of publications, percent of collaboration, and FWCI are analyzed across each of these eleven subject areas. Data related to international collaboration publication, FWCI, and the number of scholarly outputs across all the BIMSTEC countries are analyzed.

*Bangladesh*

**Figure 9.**
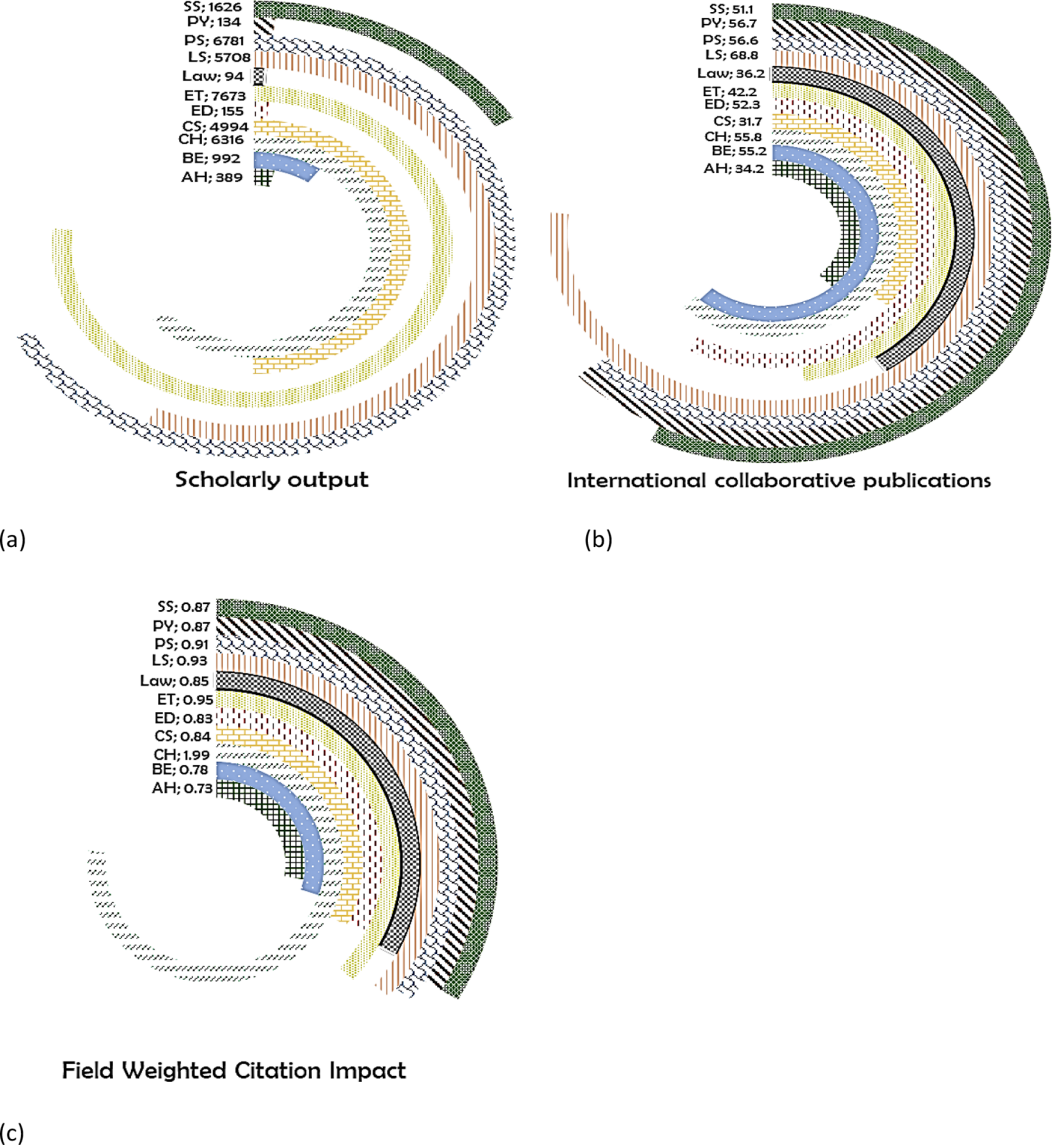
Subject wise split of Bangladesh research outcome (a) scholarly output, (b) International collaboration and (c) FWCI. Arts & Humanities (AH), Business and Economics (BE), Clinical, pre-clinical and health (CH), Computer Science (CS), Education (ED), Engineering and Technology (ET), Life Science (LS), Physical Science (PS), Psychology (PY), Social Science (SS).

Across the eleven subject areas, the maximum number of publications from Bangladesh are in the subject area of ET (7673) ( Figure 9a) with 42.2% of its publication with international collaboration ( Figure 9b) and has a FWCI of 0.95 ( Figure 9c). Around 6781 publications are in the subject area of PS with 56.6% of collaborative publication and FWCI of 0.91. In the subject area of CH there were 6316 publications with 55.8% of international coauthored publications having the highest FWCI of 1.99, which is as highest as compared to all other subject areas. In the subject area of LS, 5708 papers were published with 68.8% of it with international coauthors having a FWCI of 0.93. CS publication has a FWCI of 0.84 from 4994 publications with 31.7% of it with international collaboration. Publication share from the remaining six subject areas is minimal with SS at 1626 (FWCI, 0.87; international collaboration (IC), 51.1%), followed by BE with 992 (FWCI, 0.78; IC, 55.2%), AH with 389 (FWCI, 0.73; IC, 34.2%), ED with 155 (FWCI, 0.83; IC, 52.3%), PY with 134 (FWCI, 0.87; IC, 56.7%), and Law with 94 publications (FWCI, 0.85; 36.2%).

*Bhutan*

**Figure 10.**
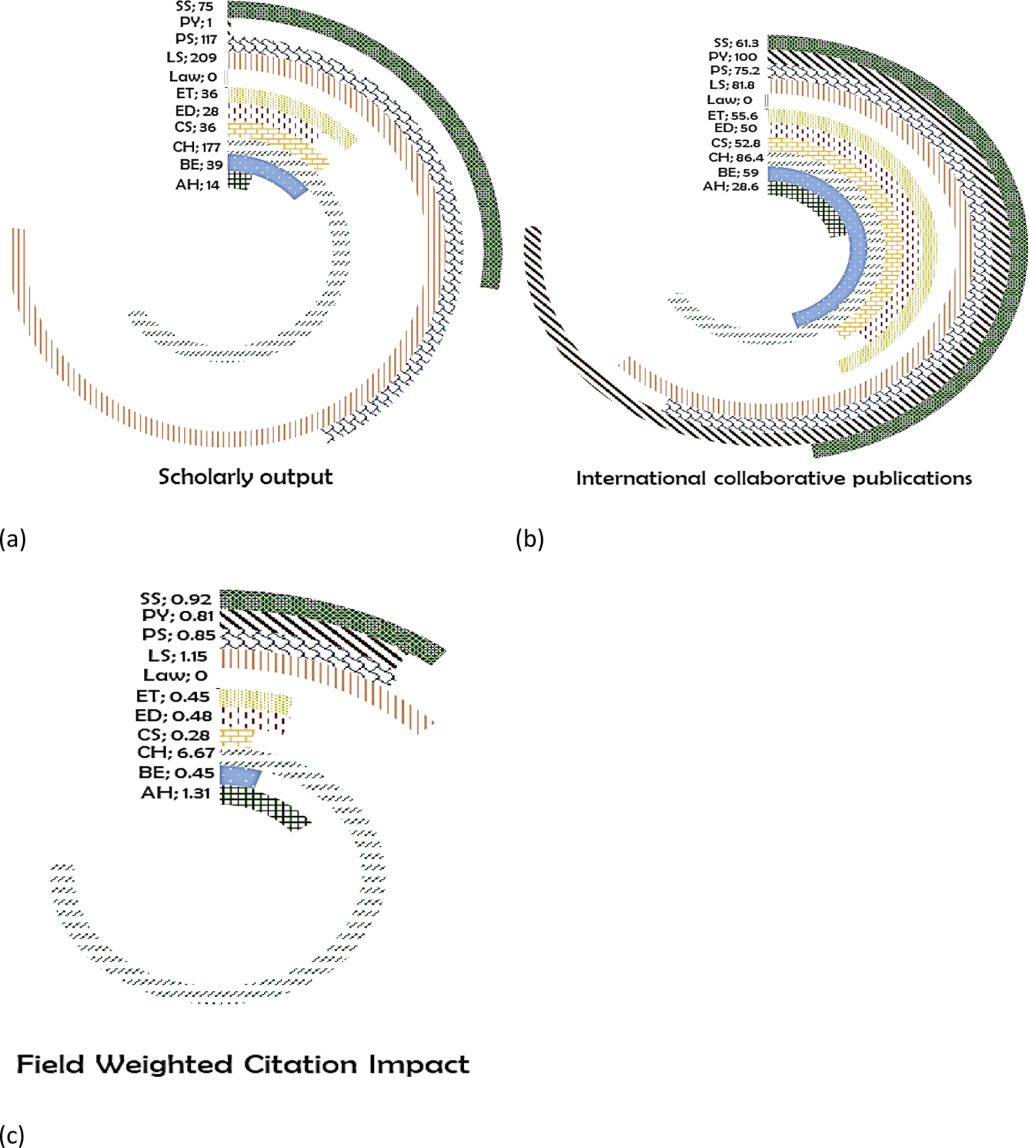
Subject wise split of Bhutan research outcome (a) scholarly output, (b) International collaboration and (c) FWCI. Arts & Humanities (AH), Business and Economics (BE), Clinical, pre-clinical and health (CH), Computer Science (CS), Education (ED), Engineering and Technology (ET), Life Science (LS), Physical Science (PS), Psychology (PY), Social Science (SS).

Across the eleven subject areas, the maximum number of publications were in the subject area LS with 209 publications ( Figure 10a), with 81.8% international collaboration ( Figure 10b), having a FWCI of 1.15 ( Figure 10c). CH has the highest FWCI in comparison to all subject areas with 6.64 having 86.4% of its publication with international coauthored from 177 publications. Around 117 publications are in the area of PS with 75.2% of them with international collaboration having a FWCI of 0.81. Publication in SS is 75 with 61.3% of it with international collaboration has a FWCI of 0.92. Publication in the area of ET and CS has an equal number of publication (36) with 55.6% (FWCI, 0.45) and 52.8% (FWCI, 0.28) of its publication with international collaboration respectively. Around 39 publications are published in the area of BE with 59% of it has international coauthored publication and FWCI of 0.45. In total, 28 papers are published in the area of ED, with 50% of its publication with international coauthors and has a FWCI of 0.48. AH with a FWCI of 1.31 has around 28.6% of its 14 publications from international authors. The subject area of PY and Law have negligible outcome in terms of publication numbers.

*India*

**Figure 11.**
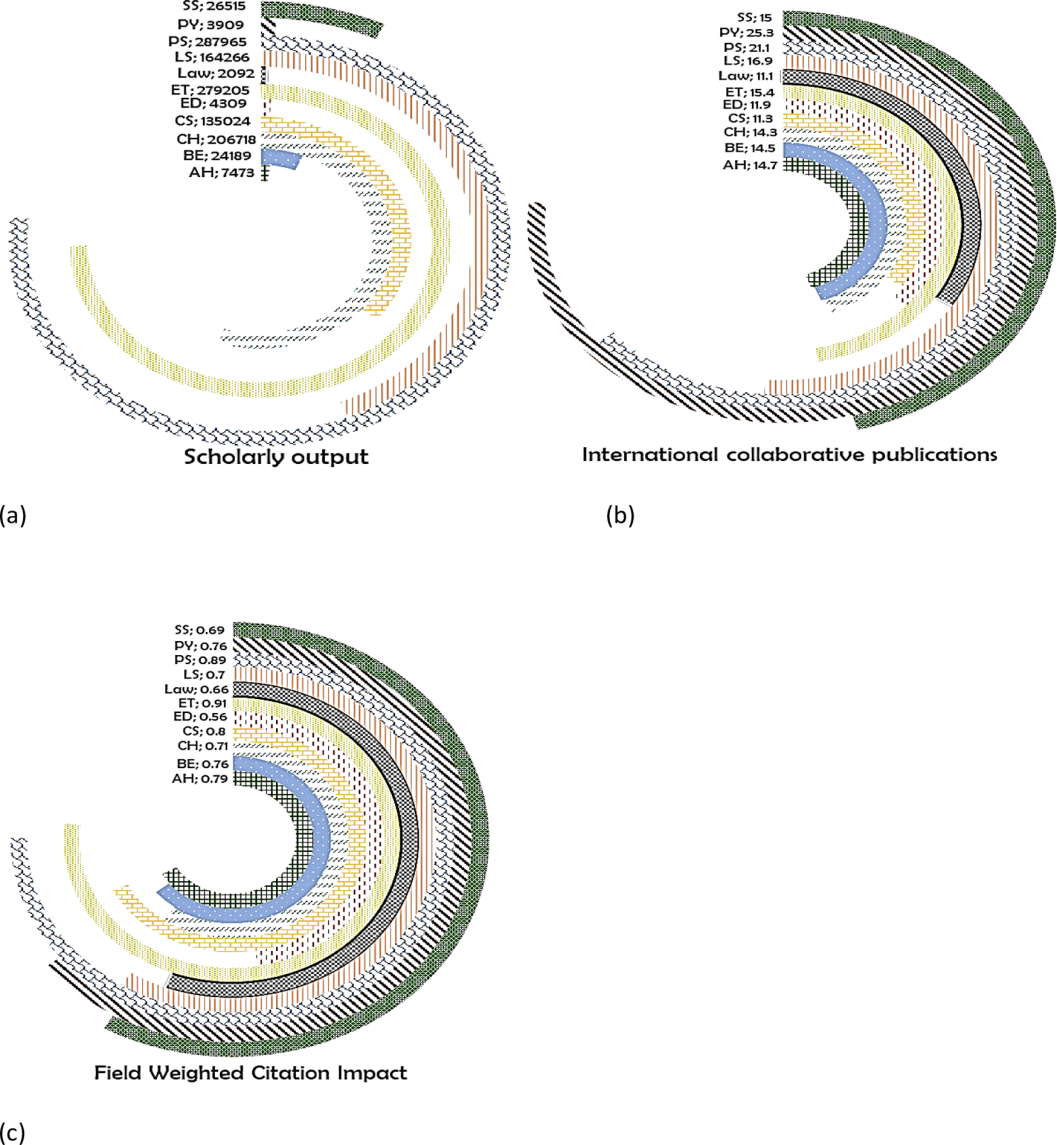
Subject wise split of India research outcome (a) scholarly output, (b) International collaboration and (c) FWCI. Arts & Humanities (AH), Business and Economics (BE), Clinical, pre-clinical and health (CH), Computer Science (CS), Education (ED), Engineering and Technology (ET), Life Science (LS), Physical Science (PS), Psychology (PY), Social Science (SS).

India produces most of its publications in the subject area of PS (287965) ( Figure 11a), with 21.1% of its publication with international collaboration ( Figure 11b) having a FWCI of 0.89 ( Figure 11c). A total of 279205 papers are published in the area of ET with 15.4% of them having international co-authorship with a FWCI of 0.91. Around 206718 papers are published in the area of CH with 14.3% of them with international authors (FWCI, 0.71). In LS, there are around 164266 publications with 16.9% international collaborative publications with a FWCI of 0.7. CS has a FWCI of 0.8 with 11.5% of the publications being co-authored by international collaborators (total, 135024 publications). SS published 26515 papers with 15% of them with international collaboration (FWCI, 0.69). In BE, there are around 24189 papers with 14.5% of them with international co-authorship (FWCI, 0.76). AH have 7473 publications 14.7% of which are internationally co-authored publications (FWCI of 0.79). In ED, there are 4309 publications with 11.9% being international co-authored with FWCI of 0.59. In total 25.3% of publications in PY out of 3909 publications are international co-authored with a FWCI of 0.76. The least number of publications are in Law with 2092 publications, 11.1% of which with international collaboration (FWCI, 0.66).

*Myanmar*

**Figure 12.**
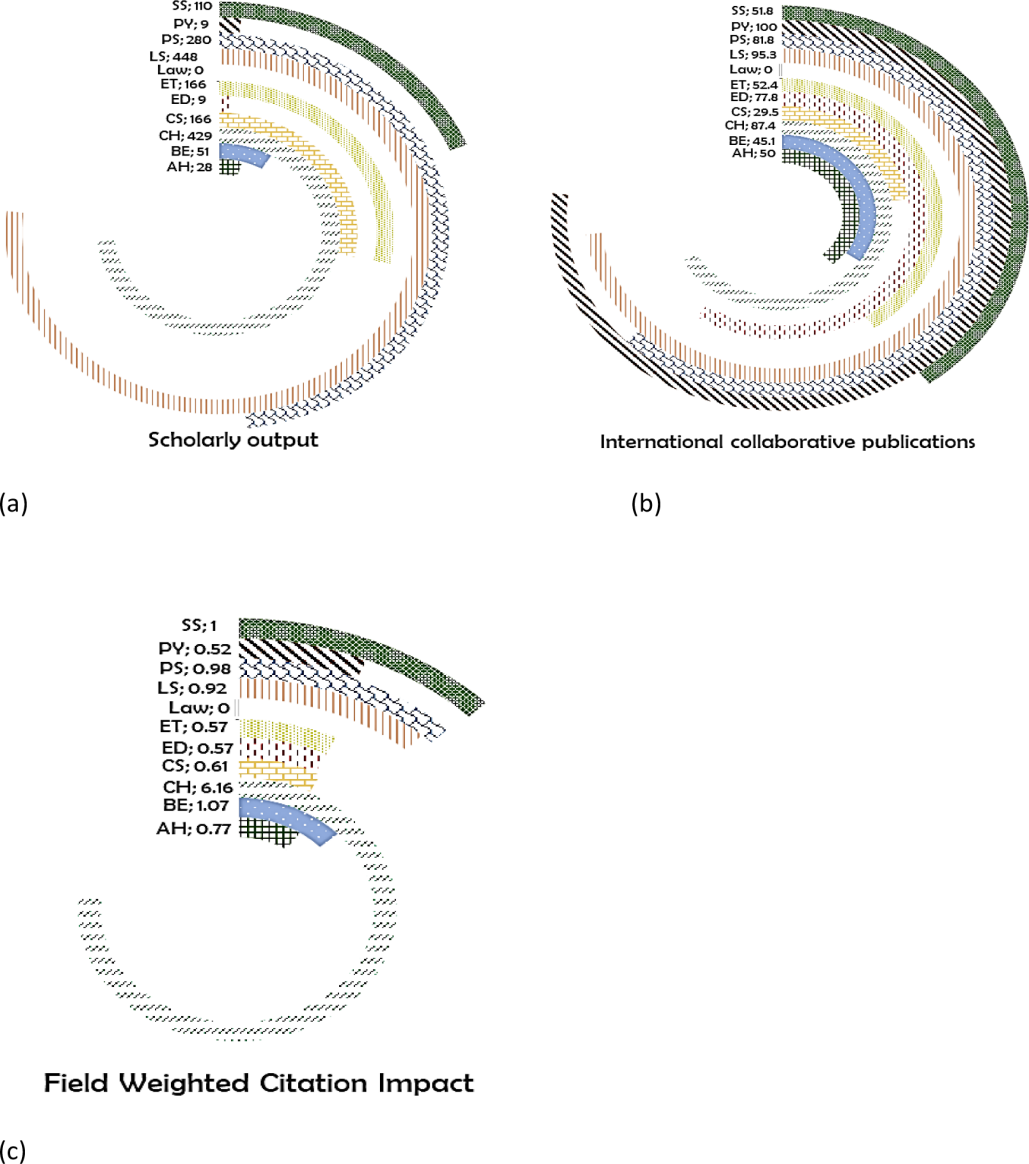
Subject wise split of Myanmar research outcome (a) scholarly output, (b) International collaboration and (c) FWCI. Arts & Humanities (AH), Business and Economics (BE), Clinical, pre-clinical and health (CH), Computer Science (CS), Education (ED), Engineering and Technology (ET), Life Science (LS), Physical Science (PS), Psychology (PY), Social Science (SS).

The maximum number of publications are in the subject area of LS (448) ( Figure 12a) with 95.3% of its publication having international coauthors ( Figure 12b), with a FWCI of 0.92 ( Figure 12c). CH (429 publications) had 87.4% of its publications with international coauthors, with FWCI of 6.16. In total, 280 papers are published in PS with 81.8% international co-authored, with FWCI of 0.98. ET have 166 publications with 52.4% international co-authored, and FWCI of 0.57. Around 166 papers are published in CS with 87.4% international coauthored, and 0.61 FWCI. Myanmar produced 110 SS papers (51.8% international collaborations; FWCI of 1.0). A total of 51 papers are published in AH, 50% of which are international coauthored (FWCI of 0.77). In ED, there are nine publications, 77.8% of which are international collaborative publications having a FWCI of 0.57. All the nine papers published in PY are with international coauthors (FWCI of 0.52). There a zero publications in the subject area of Law from Myanmar.

*Nepal*

**Figure 13.**
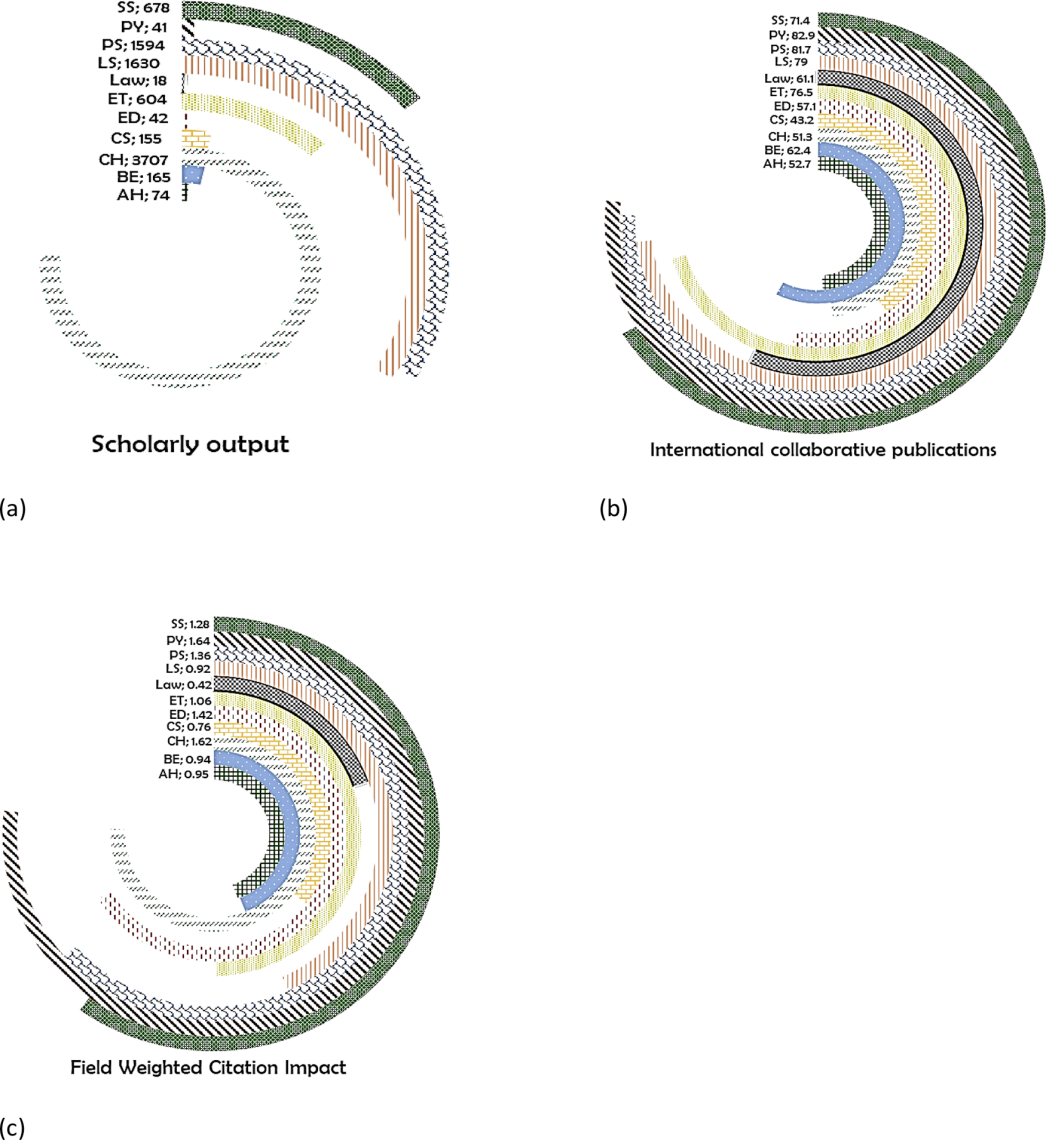
Subject wise split of Nepal research outcome (a) scholarly output, (b) International collaboration and (c) FWCI. Arts & Humanities (AH), Business and Economics (BE), Clinical, pre-clinical and health (CH), Computer Science (CS), Education (ED), Engineering and Technology (ET), Life Science (LS), Physical Science (PS), Psychology (PY), Social Science (SS).

It is seen that the highest number of publications are in the CH (3707 publications) ( Figure 13a), 51.3% of which are international coauthored ( Figure 13b), with FWCI of 1.62 ( Figure 13c). In LS, 1630 papers are published, 79% of which are international collaborative, with a FWCI of 0.92. Around 1594 papers are published in PS, 81.7% are with international collaboration (FWCI of 1.36). In SS, there are 678 publications, 71.4% international co-authored, with FWCI of 1.28. In ET, there are 604 publications (76.5% international collaboration; FWCI of 1.06). In total, 155 papers are published in CS, out of which 45.2% are international coauthored publications, having a FWCI of 0.76. In ED, 42 papers are published 57.1% of which are international collaborative, with a FWCI of 1.42. A total of 41 papers are published in PY, of which 82.9% are coauthored with international authors, having a FWCI of 1.64. The least number of papers are from Law with 18 papers, of which 61.1% are international collaborative publications having a FWCI of 0.42.

*Sri Lanka*

**Figure 14.**
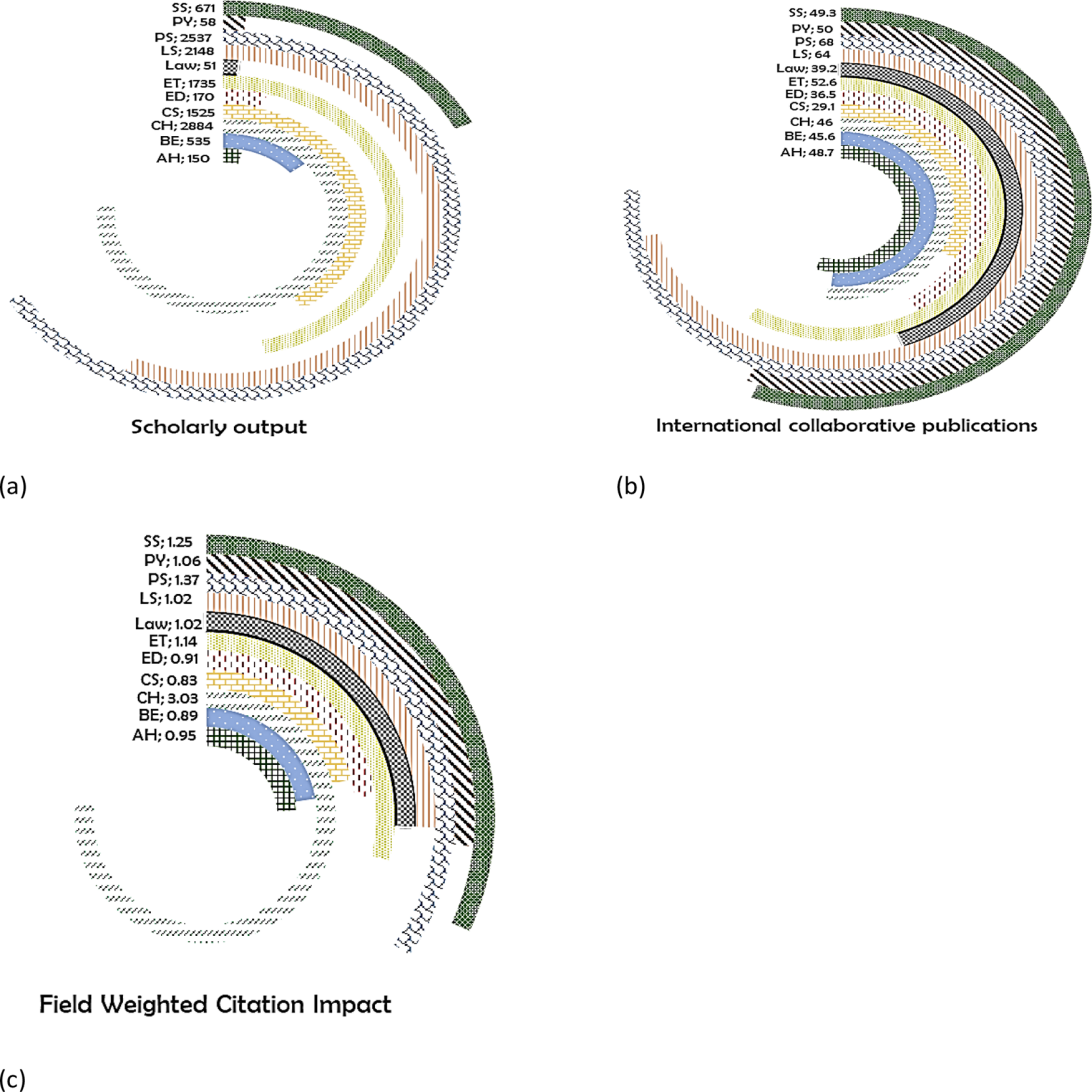
Subject wise split of Sri Lanka research outcome (a) scholarly output, (b) International collaboration and (c) FWCI. Arts & Humanities (AH), Business and Economics (BE), Clinical, pre-clinical and health (CH), Computer Science (CS), Education (ED), Engineering and Technology (ET), Life Science (LS), Physical Science (PS), Psychology (PY), Social Science (SS).

The highest number of publications for Sri Lanka is in CH (2884 publications) ( Figure 14a), 46% of its publications are with international coauthors ( Figure 14b), with a FWCI of 3.03 ( Figure 14c). In the PS, there were 2537 publications, 68% of which are with international collaboration having FWCI of 1.37. A total of 2148 papers are published in LS, with 64% international co-authored, with 1.02 FWCI. In ET, 1735 papers were published, with 52.6% with international collaboration, and a FWCI of 1.14. In CS, 1525 papers are published with 29.1% being international collaborations, with a FWCI of 0.83. A total of 671 publications in the field of SS had a FWCI of 1.25 with 49.3% of its publications with international collaborators. BE had a FWCI of 0.89 from 535 papers, with 45.6% of them being international coauthored. In ED, 170 papers are published with a FWCI of 0.91, and 36.5% of them had international collaboration. In total 150 publications are in the area of AH with 48.7% of them with international collaboration, with a FWCI of 0.95. In PY, 58 papers are published with 50% with international collaboration (FWCI of 1.06). In Law, 51 papers were published with 39.2% internationally coauthored with FWCI of 1.02.

*Thailand*

**Figure 15.**
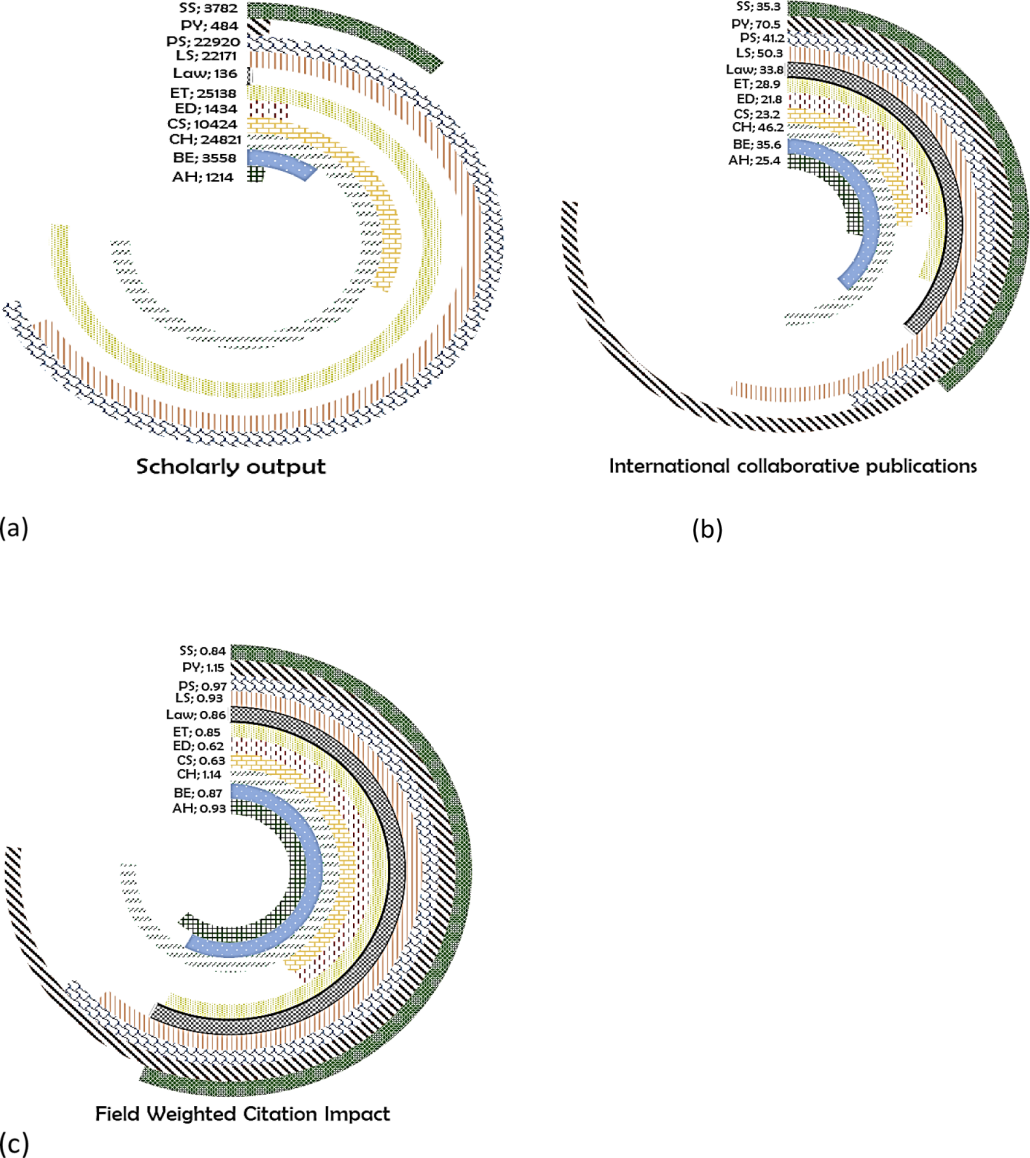
Subject wise split of Thailand research outcome (a) scholarly output, (b) International collaboration and (c) FWCI. Arts & Humanities (AH), Business and Economics (BE), Clinical, pre-clinical and health (CH), Computer Science (CS), Education (ED), Engineering and Technology (ET), Life Science (LS), Physical Science (PS), Psychology (PY), Social Science (SS).

ET have the highest number of publications for Thailand at 5138 ( Figure 15a). In total, 28.9% of these were international collaborations ( Figure 15b), with FWCI of 0.85 ( Figure 15c). In CH, there were 24821 publications with 46.2% having international co-authorship, and FWCI of 1.14. FWCI of papers published in PS is 0.97 with 22920 papers, of which 41.2% have international collaboration. In total, 50.3% of 22171 papers in LS are with international collaboration (FWCI, 0.93). In CS, 10424 papers were published with 23.2% of them international collaborations, with FWCI of 0.63. A total of 3782 papers were published in SS (35.3% international collaboration; FWCI of 0.84). In ED, 21% of papers had international coauthors, with FWCI of 0.62. In AH, 1214 papers were published with 25.4% of them international co-authored (FWCI, 0.93), while in PY, 484 papers were published with 70.5% of them international co-authored (FWCI, 1.15). The least numbers of papers were published in Law with 33.8% of 136 papers with international collaboration (FWCI of 0.86).

## Discussion

As can be seen from Table 4, Bangladesh has the highest number of papers published in the LS followed by CH. While the highest percentage of international coauthored papers are published in the field of LS, the highest FWCI is in the field of CH with ET being a close second. For Bhutan, the absolute number of papers is highest in LS, while the percentage of international coauthored publications and FWCI being highest in CH. Since only one paper in the field of PY is published with international coauthors, it is an outlier and hence not considered for analysis. The FWCI of Bhutan in CH is not only highest among all subject categories but is also highest among all BIMSTEC countries. It can be inferred that for the international community, collaborating with Bhutan is more productive in CH, with LS being a close second.

Among the BIMSTEC countries, India is demographically largest and leads in quantitative output in terms of number of publications. However, qualitatively the performance by India is poor compared with other BIMSTEC countries. The highest number of papers are published in PS, and ET, yet international coauthored papers are the highest for PY. ET has the highest FWCI, but this is lower than the world average of 1. With the highest number of HEIs established among BIMSTEC countries, a lot of researchers in India collaborate within the country. This could probably be one of the reasons for the lower percentage of international co-authored publications and lower FWCI. For Myanmar, PS is the leading subject category for publication while CH is second. A direct relationship can be established for Myanmar in terms of the number of publications and the percentage of international coauthored papers while a qualitatively higher FWCI is observed for CH. Myanmar and Bhutan show similar patterns in terms of quantitative and qualitative output. Again, papers in PY are not considered for analysis due to lower volume with all international coauthored papers.

Nepal has the highest publication output for CH and corresponding highest FWCI among all subject categories. While PY has a higher FWCI than CH, the subject category is excluded for analysis due to the relatively lower publication count skewing the data. PS has the highest number of international coauthored papers. Sri Lanka’s largest publication output is CH, with a corresponding highest FWCI whereas PS exhibits the highest international coauthored publications. In the case of Thailand, the highest papers are published in ET followed by CH. While ET has the highest papers, corresponding international coauthored papers and FWCI are relatively lower. The percentage of international coauthored papers and FWCI are highest in the subject category PY. FWCI for CH is almost equal to PY yet the difference in quantitative output is significantly larger.

From the detailed analysis of BIMSTEC countries in terms of eleven subject areas, it is observed that there is diversity in terms of publications. A few of the points perceived are:

• The least numbers of papers are published in the subject area of Law in all BIMSTEC countries.
• For all the BIMSTEC countries, CH, LS, PS, and ET constitutes a major chunk of their research output.
• Smaller countries have higher output in CH and have higher international collaboration due to various funding schemes aimed at supporting capacity building in these small countries with comparatively lower HEIs. ^1, 2^

## Conclusion

The Bay of Bengal Initiative for Multi-Sectoral Technical and Economic Cooperation (BIMSTEC) is an international organization of seven nations of South Asia and Southeast Asia. BIMSTEC was an initiative to accelerate economic growth and social progress. BIMSTEC also promotes partners to have active collaboration and excel in technical and scientific fields. The progress in a scientific field is usually measured in terms of publication, grant, and innovation. The objective of this paper was to analyze the publication output of these seven nations using various bibliometric parameters.

Demographically and geographically larger countries like India and Thailand among BIMSTEC have better quantitative research outcomes. However, smaller countries like Bhutan, Myanmar, Sri Lanka, and Nepal have better qualitative outcomes such as percentage of international coauthored papers and FWCI While subject categories of Clinical, preclinical and Health, Physical Science, and Engineering and Technology are the focus areas of research, Arts and Humanities, Psychology, Education and Law are less preferred.

## Data availability

Open Science Framework: BIMSTEC, https://doi.org/10.17605/OSF.IO/4D32C. ^22^

Data are available under the terms of the Creative Commons Attribution 4.0 International license (CC-BY 4.0).
