# Supplementary material for: Publication analysis in Bay for Bengal Initiative for Multi-Sectoral Technical and Economic Cooperation Nations
Source: F1000Res. 2021 Jun 28;10:510. [Version 1] doi: 10.12688/f1000research.52286.1 (PMC8619851; doi:10.12688/f1000research.52286.1)
Supplement: Peer review history

# Peer review: Reviewer response for version 1

**DOI:** 10.5256/f1000research.55540.r97163

**Published:** 2021-11-25

**Authors:** Samile Andréa de Souza Vanz

**Affiliations:** 1 Federal University of Rio Grande do Sul (UFRGS), Porto Alegre, Brazil

The paper presents an interesting historical background of seven BIMSTEC countries which is very useful to understand the context of each country. Publication output from Bangladesh, India, Myanmar, Sri Lanka, Nepal, Thailand, and Bhutan is obtained for the period 2012 to 2017 from Scopus, a great database to identify papers published around the world. As an international database, Scopus is not specific and embracing for each country, but permits a comparison between nations. The World Bank Indicators are also adequate to compare different nations.

- Data from SciVal coincide with the period 2021-2017 and the Scopus dataset period is 2012-2017. It is important to explain why a different period was chosen, why and when Scopus and SciVal data were used.
- The descriptive analysis presented, figures and tables are very illustrative.
- The results and discussion could improve with a review of previous bibliometric studies, especially those related to the 7 countries.
- Another important ponderation must be the volume of journals indexed by Scopus. Everyone knows that it is a very selective database. I suggest the authors mention how many journals from each country are indexed compared to how many are published by each country. The output indexed by Scopus is just a part of the total produced by each country.

Is the work clearly and accurately presented and does it cite the current literature?

Partly

If applicable, is the statistical analysis and its interpretation appropriate?

Not applicable

Are all the source data underlying the results available to ensure full reproducibility?

Yes

Is the study design appropriate and is the work technically sound?

Yes

Are the conclusions drawn adequately supported by the results?

Partly

Are sufficient details of methods and analysis provided to allow replication by others?

Yes

Reviewer Expertise:

Bibliometrics, scientific collaboration.

I confirm that I have read this submission and believe that I have an appropriate level of expertise to confirm that it is of an acceptable scientific standard.

# Peer review: Reviewer response for version 1

**DOI:** 10.5256/f1000research.55540.r91025

**Published:** 2021-08-06

**Authors:** Shankar Reddy Kolle

**Affiliations:** 1 Indira Gandhi National Tribal University, Amarkantak, Madhya Pradesh, India

The work entitled "Publication analysis in Bay for Bengal Initiative for Multi-Sectoral Technical and Economic Cooperation Nations" was carried out using the data from the Scopus database and analyzed using the SciVal tool in terms of scholarly output, field-based citations impact, collaboration with other countries, subject-wise contribution, along with international collaboration. The study's significant findings are that more significant countries like India and Thailand have contributed immensely than smaller countries. However, a more detailed analysis of the number of papers in the top ten high-impact factor journals would be helpful to see the research progress in the BIMSTEC countries. This work would be helpful to know for researchers for the research contribution of BIMSTEC countries.

Is the work clearly and accurately presented and does it cite the current literature?

Yes

If applicable, is the statistical analysis and its interpretation appropriate?

Yes

Are all the source data underlying the results available to ensure full reproducibility?

Yes

Is the study design appropriate and is the work technically sound?

Yes

Are the conclusions drawn adequately supported by the results?

Yes

Are sufficient details of methods and analysis provided to allow replication by others?

Yes

Reviewer Expertise:

Bibliometric analysis, scientometric analysis, research evaluation.

I confirm that I have read this submission and believe that I have an appropriate level of expertise to confirm that it is of an acceptable scientific standard.
